# Perspectives of human verification via binary QRS template matching of single-lead and 12-lead electrocardiogram

**DOI:** 10.1371/journal.pone.0197240

**Published:** 2018-05-17

**Authors:** Vessela Krasteva, Irena Jekova, Ramun Schmid

**Affiliations:** 1 Institute of Biophysics and Biomedical Engineering, Bulgarian Academy of Sciences, Sofia, Bulgaria; 2 Signal Processing, Schiller AG, Baar, Switzerland; Indiana University, UNITED STATES

## Abstract

**Objective:**

This study aims to validate the 12-lead electrocardiogram (ECG) as a biometric modality based on two straightforward binary QRS template matching characteristics. Different perspectives of the human verification problem are considered, regarding the optimal lead selection and stability over sample size, gender, age, heart rate (HR).

**Methods:**

A clinical 12-lead resting ECG database, including a population of 460 subjects with two-session recordings (>1 year apart) is used. Cost-effective strategies for extraction of personalized QRS patterns (100ms) and binary template matching estimate similarity in the time scale (matching time) and dissimilarity in the amplitude scale (mismatch area). The two-class person verification task, taking the decision to validate or to reject the subject identity is managed by linear discriminant analysis (LDA). Non-redundant LDA models for different lead configurations (I,II,III,aVF,aVL,aVF,V1-V6) are trained on the first half of 230 subjects by stepwise feature selection until maximization of the area under the receiver operating characteristic curve (ROC AUC). The operating point on the training ROC at equal error rate (EER) is tested on the independent dataset (second half of 230 subjects) to report unbiased validation of test-ROC AUC and true verification rate (TVR = 100-EER). The test results are further evaluated in groups by sample size, gender, age, HR.

**Results and discussion:**

The optimal QRS pattern projection for single-lead ECG biometric modality is found in the frontal plane sector (60°-0°) with best (Test-AUC/TVR) for lead II (0.941/86.8%) and slight accuracy drop for -aVR (-0.017/-1.4%), I (-0.01/-1.5%). Chest ECG leads have degrading accuracy from V1 (0.885/80.6%) to V6 (0.799/71.8%). The multi-lead ECG improves verification: 6-chest (0.97/90.9%), 6-limb (0.986/94.3%), 12-leads (0.995/97.5%). The QRS pattern matching model shows stable performance for verification of 10 to 230 individuals; insignificant degradation of TVR in women by (1.2–3.6%), adults ≥70 years (3.7%), younger <40 years (1.9%), HR<60bpm (1.2%), HR>90bpm (3.9%), no degradation for HR change (0 to >20bpm).

## 1. Introduction

Since the early 2000s, the electrocardiogram (ECG) has been suggested as a biometric modality for human identity recognition [1–5]. The main concern is the use of a low-cost and routine acceptable physiological measurement, providing a unique behavioral characteristic that is always present for robust liveness detection in secure authentication and access control systems. Since 2008, special attention is given to the simplest user interface for unobtrusive “off-the-person” technique for single-lead ECG acquisition via two Ag/AgCl electrodes at the hands or fingers [6–11] with the closer proximity to deployable real-life biometric applications. The most recent developments on the state-of-the-art biometric technologies utilize ECG-based authentication algorithms in: remote healthcare monitoring scenarios [12, 13] with biosensors integrated into mobile devices [14, 15]; wearable smart watch-type devices [16]; secure wireless body area sensor networks [17–19]; continuous authentication applications with adaptive strategies for tracking of the individual beat variations in 24h ECG recordings [20]; short-term authentication applications for patient validation support and error screening of digital hospital databases with multi-session conventional (10s, 12-lead) ECG recordings [21, 22]. The multi-lead scenarios for biometric recognition are proposed for improving of the authentication accuracy. We find fewer studies for comparative investigation of the optimal single or multi-lead ECG combination schemes [1, 21–26]. In one aspect, it is important to achieve position invariant measurements by recording ECG signals from the three leads fixed to the extremities, according to the Einthoven’s triangular scheme, shown to be widely independent of the actual positioning of the electrodes [27]. However, alternative leads from electrodes on the human chest, close to the heart source, are shown to be more informative for the discrimination between individuals [23], although not confirmed in [21, 22], probably due to database differences.

An actual limitation of the ECG-based biometrics is the lack of standardization for benchmarking the performance in an objective way due to unavailable exhaustive ECG biometric databases on the public repository [28]. Numerous biometric studies use public clinical databases from the Physionet databank [29] or private sources with customized protocol for data collection, employing comparison of sequences of beats from a single-session ECG recording per subject [3, 14, 15, 17–20, 23, 30–34]. These single-session studies could track the inherent variations of different heartbeats in the same subject, but miss the intra-subject variability of the beat morphology due to physiologically related long-term ECG changes (over months and years) or potential misplacement of the electrodes from their anatomical landmarks across different sessions. Other studies are designed on small-sized databases (<30 subjects), thus missing the statistical validation of the inter-subject variability across a large population [1, 2, 7–9, 13, 16–19, 24, 32, 35–37].

This study aims to present a new cost-effective strategy for 12-lead ECG-based biometrics, which compares the beat morphologies of two individuals by binary template matching of short-duration QRS patterns (100ms). The aim is to capture a minimal feature set, including only two straightforward QRS pattern characteristics per lead, named similarity in the time scale (matching time) and dissimilarity in the amplitude scale (mismatch area). An unbiased validation of those features for the aims of human verification, applying linear discriminant statistical analysis of one-year distant measurements over an uncommonly large population, is an important asset to provide further evidence about the stability and scalability of the ECG as a biometric modality. The statistical analysis is presented for different perspectives of the human verification problem, i.e. the choice of the optimal single and multi-lead ECG set; the influence of the test database size and different physiological factors (gender, age, heart rate).

## 2. Related studies on QRS template matching

Although the great number of recent studies on ECG biometrics with evidence in extensive literature surveys and reviews [12, 30, 38–40], this field is still in the state of active research on different ECG transforms, extracted features and classification methods. We further review different template matching techniques, which utilize the biometric information carried by the beat morphology. Generally, the template matching process involves: pre-processing, template extraction, feature calculation, dimensionality reduction and classification.

- Pre-processing: A narrow band-pass filter (low/high cut-off frequencies in the range (0.5-5Hz)/(15-100Hz)) is a crucial pre-processor for the ‘Off-the-person’ ECG acquisition via Ag/AgCl electrodes at the hands or fingers [6–11] that is much more prone to noise than the regular ‘On-the-person’ electrodes with conductive paste or gel interface.
- Template extraction: It relies on QRS fiducial point detection, followed either by P-QRS-T segmentation [7, 10, 11, 14, 35, 36, 41, 42] or fixed window selection [8, 9, 13, 19, 20, 22, 24, 32, 35, 43, 44]. The periodicity transform, using a segmented autocorrelation function [45] or a short-time Fourier transform (STFT) within the selected window [9, 46] has also been effectively employed for beat pattern representation. The noise immunity of the extracted templates is improved by different techniques: outlier removal of irregular or low-quality beats [8, 10, 11, 43], heartbeat alignment [10, 11, 13, 14, 22, 44], signal-averaging of consecutive beats [8, 10, 22, 24], spline interpolation of beats [13], amplitude normalization to mitigate the effect of intra-subject amplitude variations [7, 13, 32, 44]; continuous template update [20]. The intra-subject heart rate dependent variations of the heartbeat are typically compensated by normalization of the QT interval and related temporal features to the momentous RR interval by linear [3, 14, 35, 22, 42] and non-linear [42] correction transforms, as well as PQRST decimation to fixed length [7, 35]. Although the importance of QT correction has been clearly demonstrated by preventing degradation of identification rate over time, no clear choice among seven explored approaches for QT correction has been recommended [42], as well as non substantial profit (<0.4%) has been found for QT correction by fixed length vs. Framingham’s formula [35].
- Feature calculation: The commonly calculated template matching features are: cross-correlation coefficients [3, 6, 11, 16, 20, 22, 24, 25, 33, 41], autocorrelation [11, 13], Euclidean distance [7, 11, 34, 43, 44], Mahalanobis distance [23], cosine distance [8], percent residual difference [6], wavelet distance [6, 15], weighted distance with the inverse mutual quality [45], log-likelihood ratio [9, 45], higher order statistics [19].
- Dimensionality reduction: The dimensionality reduction techniques are based on principal component analysis (PCA) [10, 23, 34, 36, 37, 44], linear discriminant analysis (LDA) [22, 37, 45], fast Fourier transform [11], Hermite polynomials expansion coefficients [32], discrete cosine transform coefficients [35], singular value decomposition [19], ensemble empirical mode decomposition [34], information-gain ratio (IGR) [37], parameterized averaged support heuristics (PASH) algorithm [37], symmetric relative entropy for selection of features with distinguishability and stability [9, 46].
- Classification: The classification algorithms used in the template matching studies apply K-nearest neighbours (k-NN) [7, 8, 11, 13, 15, 34, 43], Bayesian classifiers [13, 23, 36], support vector machines (SVM) [8, 32], decision-based neural networks (DBNN) [3, 35], random forest [15], constant or probabilistic threshold rules [9, 15, 16, 19, 20, 24, 25, 33, 44–46].

## 3. Materials and methods

### 3.1. Database

This retrospective study considers a proprietary clinical ECG database, provided with the courtesy of Schiller AG (Switzerland) for the purpose of human biometrics on a large population observed over time:

- Recording place: Emergency Department of the University Hospital Basel (2004–2009)
- Population: 460 non-cardiac patients (235/225 male/female, 18–106 years old)
- Recordings: 10s resting ECG, standard 12-leads
- Sessions: Two sessions per subject, recorded at distant time points (>1 year)
  ○ First (reference) session: S1
  ○ Second (remote) session: S2>S1+1 year
- ECG device: Commercial SCHILLER AT-110 for digital recording of 12-lead ECG with resolution (500Hz, 2.5μV/LSB). The ECG is filtered in a diagnostic bandwidth by high-pass (0.05Hz) and low-pass (150Hz) first order analog filters (20dB/decade).
- Anonymization: The biometric database is anonymized and analyzed under conditions keeping the privacy of the involved subjects.

**Fig 1:**
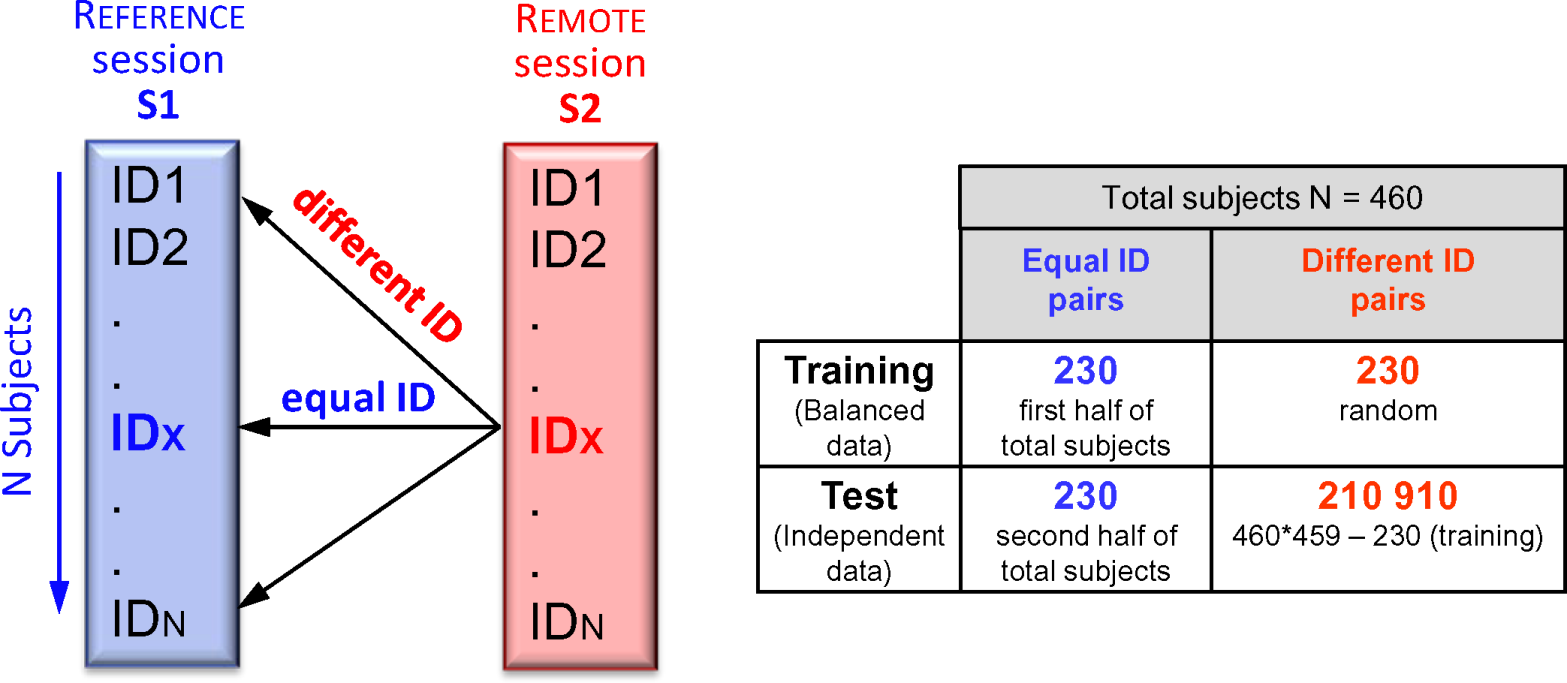
Scheme for comparison of subjects between S1 and S2 sessions. Content of the training and test datasets considering all pairwise ID combinations (S2 vs. S1).

The person verification scheme for comparison of subjects between S1 and S2 sessions gives a total of N = 460 pairs of subjects with equal identity (ID) and N*(N-1) = 211140 pairs of subjects with different ID. Our approach to handle the imbalance ratio (459:1) of different-to-equal ID pairs considers two independent datasets (Fig 1):

- Training dataset: 230/230 equal/different ID pairs, presuming that the verification classifier should be trained on the first half of subjects using balanced data, not over-fitted to any of the classes.
- Test dataset: 230/210910 equal/different ID pairs, ensuring that unbiased classifier performance is further reported on a big dataset, including all available cases fully independent from the training.

### 3.2. QRS pattern analysis

The presented method for extraction of subject-specific ECG information is focused on the QRS waveform, being a prominent feature in many heartbeat classification and automated diagnostic system. Besides, we consider the stability of the QRS complex to the heart rate, previously proved to outperform the QT-signal for the purpose of ECG biometrics [35]. The main methodological concern is the proper extraction of 12-lead QRS patterns and the subsequent quantification of the lead-specific QRS waveform differences between pairs of recordings. It is presented below as a four-stage QRS pattern analysis process, including: (1) QRS pattern extraction; (2) amplitude normalization; (3) time-amplitude approximation; (4) pattern matching and feature extraction.

#### 3.2.1. QRS pattern extraction

**Fig 2:**
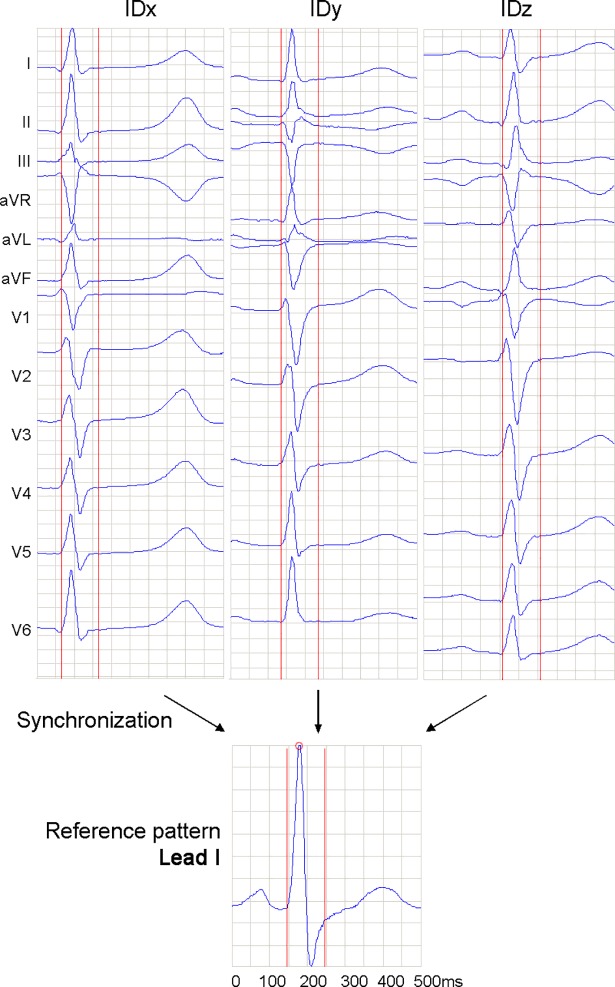
Example of 12-lead average beat patterns from three different subjects (with identity named IDx, IDy, IDz), which are aligned by maximal cross-correlation in lead I to a reference pattern. The vertical red lines encompass the synchronously extracted 12-lead QRS pattern in a window [-30ms; 70ms] around the R-peak of the reference pattern.

Each ECG recording is processed by a certified commercial ECG measurement and interpretation module (ETM, Schiller AG, Switzerland) for extraction of a 12-lead average beat with duration of 500ms. The embedded arrhythmia detection and lead quality monitoring algorithms reject beats with abnormal morphologies (e.g. ventricular extrasystoles and artifacts). The average beats are commonly used for measurement of ECG waves with diagnostic precision because they provide higher signal-to-noise ratio (SNR) and are more robust with respect to respiration induced morphology changes than the single beats. We observe a time shift between the average beats from different recordings (Fig 2). Therefore, the task for extraction of aligned QRS patterns is of crucial importance for the correct inter-subject comparisons. In order to provide a more accurate analysis during the subsequent time-alignment and QRS pattern matching calculations, the time resolution of the average beats is increased to 1 ms by resampling from 500 to 1000 Hz. We employ the Matlab function ‘resample’ (upsampling with a Kaiser window anti-aliasing filter). The time-alignment is performed by maximal cross-correlation between the average beat and a reference pattern. The reference pattern (Fig 2) has been initialized at the beginning of the study as a ‘normally’ behaving average beat in lead I (with positive P-QRS-T waves), belonging to a subject from the population.

At the next step, the QRS patterns of all subjects are synchronously extracted for all 12-leads, taking the subject’s average beat in a window of 30ms before and 70ms after the fiducial point, aligned to the R-peak of the reference pattern (see Fig 2). The window length of 100ms was not tuned with respect to the specific biometric study, rather it reflects the average length of a normal QRS interval. The short window protects the selected pattern to include the P, T-waves and ST-interval, taking into consideration the findings of our previous study [21]. It distinguished the biometric potential of the amplitude-temporal features of R, S-waves and rejected P, ST, T parts due to low intra-subject reproducibility and low inter-subject variability. This is also confirmed in [44], reporting that P-waves are dominated by noise, while T-waves are not distinct for biometrics.

#### 3.2.2. QRS pattern amplitude normalization

In order to compensate for large inter-subject and inter-lead amplitude spans, the amplitudes of 12-lead QRS patterns in any ID pair from sessions Si = (*S*1, *S*2) and lead Li = (1, 2,.., 12) are linearly scaled to fit in the range [-1;1]. For this purpose, each lead of the QRS pattern $QRS_{Li}^{Si}(ti)$ is first shifted such that the QRS onset (determined by ETM) lies at 0 V. Then $QRS_{Li}^{Si}(ti)$ is divided by a scale factor, equal to the maximal absolute amplitude over time *ti* = (1-100ms) among S1 and S2 sessions:

$$QRS_{Li}^{Si}(ti)=\frac{QRS_{Li}^{Si}(ti)}{\underset{Si=S1,S2}{\max}\;\underset{\mathrm{ti}=1-100\mathrm{ms}}{\max}abs(QRS_{Li}^{Si}(ti))}\in [-1;1].$$ (1)

The aim of normalization is to further use the same computational range [-1;1] for all individuals, regardless of their signal amplitudes. We note that the re-scaling process is offline and there is no need for any prior settings of the scale factor based on unknown (expected) amplitudes.

#### 3.2.3. QRS pattern time-amplitude approximation

The one-dimensional vector of the QRS pattern over time $QRS_{Li}^{Si}(t)$ is transformed to a 2D binary matrix $binQRS_{Li}^{Si}(t,a)$, applying the following approximation:

$$binQRS_{Li}^{Si}(ti,aj)=\{\begin{array}{ll} 1 & \mathrm{if}\;A(aj)\in [QRS_{Li}^{Si}(ti)\pm 2\Delta a]\;\mathrm{or}\;A(aj)\in [QRS_{Li}^{Si}(ti\pm \Delta t)]\\ 0 & \mathrm{otherwise} \end{array},$$ (2)

where:

- ti is the index of the columns, representing the time grid: T(ti) = [1,1+Δt,1+2Δt,…,100] ms with resolution Δt.
- aj is the index of the rows, representing the amplitude grid: A(aj) = [-1,-1+Δa,-1+2Δa, …,1] with resolution Δa.

Larger values of (Δt, Δa) form a coarse grid, which makes a more rough approximation of $QRS_{Li}^{Si}(t)$ in a smaller size $binQRS_{Li}^{Si}(t,a)$ matrix at the cost of potential loss of QRS pattern waveform details. In contrary, smaller values of (Δt, Δa) form a fine grid, which makes a more fine approximation of $QRS_{Li}^{Si}(t)$ in a larger size $binQRS_{Li}^{Si}(t,a)$ matrix, thus increasing the computation cost. In our application, the settings of both resolutions are:

- Δt = 1 ms delineates the finest resolution in the time-scale, defined by the sampling rate of 1000 Hz.
- Δa = 0.025 (normalized units) is equivalent to Δa = 1.25% in the amplitude scale range [-1;1].

**Fig 3:**
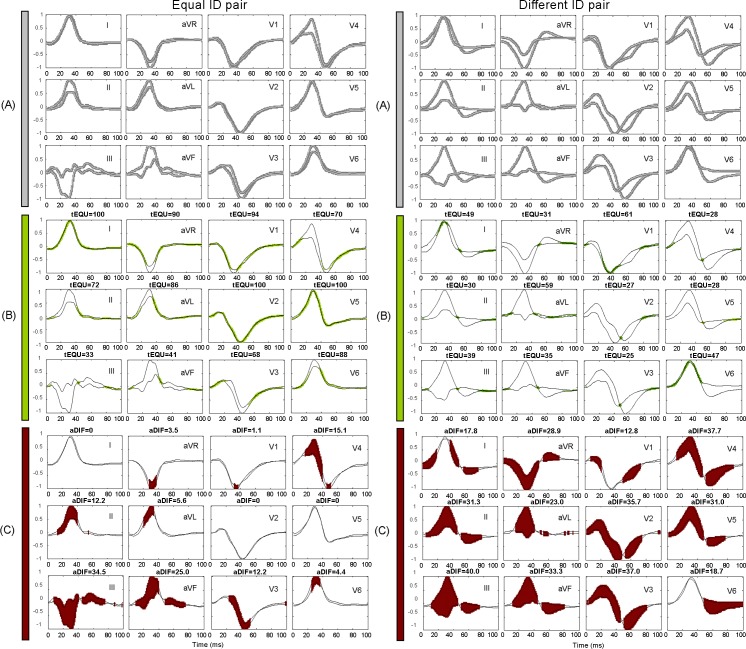
Example of 12-lead QRS pattern matching between S1 and S2 sessions, taking recordings from equal ID subjects (left panel) and different ID subjects (right panel). (A) The grey approximation span around each QRS pattern (white trace) represents the ones in the corresponding 2D binary matrix binQRS(100x80). (B) The green zones represent the ones in the binary AND matrix for computation of tEQU, matching the time equivalence between the two patterns (black traces). (C) The red elements represent the ones in the binary NAND matrix for computation of aDIF, matching the area difference between the two patterns (black traces).

The size of the binary matrix $binQRS_{Li}^{Si}$ is 100 columns and 80 rows, occupying a memory of 1kB per lead. On demand, it can be easily re-sized by changing (Δt, Δa) settings. The present settings equalize small variations of the cardiac depolarization process within ±Δt (±1ms) over time and ±2Δa (±2.5%) over amplitude, as defined in the approximation transform (Eq 2). Fig 3A illustrates the approximation span around $QRS_{Li}^{Si}$, while it is reproduced in the binary matrix $binQRS_{Li}^{Si}(100x80)$ for 12 ECG leads (*Li* = 1, 2,.., 12) and two recording sessions (Si = S1, S2). For most of the leads, the approximation spans (gray area) are considerably overlapped for QRS patterns from the same subject (left side) and substantially distinct for different subjects (right side).

#### 3.2.4. QRS pattern matching and feature extraction

Simple binary matching operations are applied on the matrices $binQRS_{Li}^{S1}$ and $binQRS_{Li}^{S2}$ to quantify the lead-specific similarity of the QRS pattern waveforms between S1 and S2 sessions by means of two measures:

- Time equality measure (tEQU) counts the time for overlapping of both QRS patterns after binary element-wise multiplication (AND operation) of $binQRS_{Li}^{S1}$ and $binQRS_{Li}^{S2}$:
  $$tEQU_{Li}\{S1,S2\}=\sum_{ti=1}^{100}binQRS_{Li}^{S1}(ti,aj)\wedge binQRS_{Li}^{S2}(ti,aj)$$ (3)
  $$tEQU_{Li}\{S1,S2\}=\frac{\Delta t}{100ms}*tEQU_{Li}\{S1,S2\}*100\in [0;100],\%$$ (4)
  where *Li* = (1–12), aj = (1–80). Scaling by the time resolution (Δt) gives a normalized tEQU value that could be further easily interpretable, where 100% corresponds to full-time coincidence, i.e. patterns have at least one overlapping *binQRS* entry per time step (1 ms), and 0% corresponds to null coincidence, i.e. patterns do not overlap for any *binQRS* entry over the complete pattern length.
- Area difference measure (aDIF) counts the area enclosed between the non-overlapping amplitudes of both QRS patterns after binary element-wise multiplication and inversion (NAND operation) of $binQRS_{Li}^{S1}$ and $binQRS_{Li}^{S2}$:
  $$aDIF_{Li}\{S1,S2\}=\sum_{ti=1}^{100}\sum_{aj=a\min}^{a\max}binQRS_{Li}^{S1}(ti,aj)\bar{\wedge }binQRS_{Li}^{S2}(ti,aj),$$ (5)
  where the summation interval in the amplitude scale is enclosed between the minimal and maximal QRS amplitudes among S1 and S2 patterns, measured at each specific time index ti, i.e. $\left[a\min(ti)=\underset{Si=S1,S2}{\min}binQRS_{Li}^{Si}(ti\right)$; $a\max(ti)=\underset{Si=S1,S2}{\max}binQRS_{Li}^{Si}(ti)$].

$$aDIF_{Li}\{S1,S2\}=\frac{\Delta t}{100ms}*\Delta a*aDIF_{Li}\{S1,S2\}*100\in [0;100],(\%)$$ (6)

Scaling by the time (Δt) and amplitude (Δa) resolution gives a normalized aDIF value that could be further easily interpretable, where 0% corresponds to full-amplitude coincidence, i.e. patterns overlap for all *binQRS* entries over the complete pattern length, and 100% corresponds to pattern differences that cover the full amplitude range, i.e. all *binQRS* entries.

For better comprehension, the resultant matrices from the binary AND and NAND operations and the respective values of tEQU and aDIF measures are illustrated in the examples of Fig 3B and 3C after matching of 12-lead QRS patterns of equal and different ID subjects.

A total set of 24 features (12-leads x 2 features per lead (tEQU_Li_, aDIF_Li_)) is defined to quantify the QRS pattern differences. Their numerical measurements over the whole population are provided within the supporting information file (S1 File). The signal-processing and feature measurement scheme is implemented in Matlab (The Mathworks Inc.).

### 3.3. Human verification model

The human verification task answers the question: “*Is the subject who he/she claims to be*?*”*. The designed human verification model takes the binary decision ‘verified’ or ‘rejected’ subject ID, comparing pairs of ECG recordings {S1,S2} by means of LDA classifier with input feature vector:

$$XLDA_{Li}=[tEQU_{Li}\{S1,S2\},aDIF_{Li}\{S1,S2\}],$$ (7)

where Li = (1–12) is the set of leads involved in the analysis.

The human verification performance is estimated with the statistical indices:

- True acceptance rate: $TAR=\frac{\mathrm{Number}\;\mathrm{Correct}\;\mathrm{Verifications}}{{\mathrm{Nb}\;\mathrm{Comparisons}\;(\mathrm{ID}}_{S1}={\mathrm{ID}}_{S2})}.100,(\%)$
- True rejection rate: $TRR=\frac{\mathrm{Number}\;\mathrm{Correct}\;\mathrm{Rejections}}{{\mathrm{Nb}\;\mathrm{Comparisons}\;(\mathrm{ID}}_{S1}\neq {\mathrm{ID}}_{S2})}.100,(\%)$
- True verification rate: $TVR=\frac{TAR+TRR}{2},(\%)$

where TAR is calculated for all equal identity pairs (ID_S1_ = ID_S2_), TRR is calculated for all different identity pairs (ID_S1_≠ID_S2_), and TVR (the common mean of TAR and TRR) is reported to equally weight both acceptance and rejection rates in an unbalanced data with number of comparisons (ID_S1_ = ID_S2_) << (ID_S1_≠ID_S2_), seen in the test dataset (defined above in section Database).

We note that part of the biometric studies report their accuracy in terms of false acceptance rate (FAR), false rejection rate (FRR) and equal error rate (EER), where EER is valid for FAR = FRR. There is a straightforward relationship between both kinds of results, which could be recalculated by the direct conversion: FAR = 100-TAR, FRR = 100-TRR, EER = 100-TVR (valid for TAR = TRR). We further interpret our accuracy results in terms of positive merit maximization (TAR, TRR, TVR), instead of negative error minimization (FAR, FRR, EER).

Non-redundant LDA models are trained by stepwise feature selection until maximization of the area under the receiver operating characteristic curve (ROC AUC). The ROC is calculated by changing the operating LDA threshold function through scanning the full-range of prior-probabilities of equal-to-different identity pairs (ID_S1_ = ID_S2_):(ID_S1_≠ID_S2_)∈[0;1], using only samples from the training database. We use the test database, fully independent of the training, to finally report the test ROC as unbiased estimation of the human verification model’s performance.

### 3.4. Statistical study

The statistical study is presented for different perspectives of the human verification problem: comparative study of single and multi-lead ECG configurations, influence of the test database size and different physiological factors (gender, age, heart rate). The Statistics toolbox in Matlab (The Mathworks Inc.) has been used for management of the statistical study, including training and evaluation of the forward stepwise LDA models. The non-normal features distributions (tEQU and aDIF, represented as median value, quartile range) are compared via the non-parametric Wilcoxon signed-rank test. The comparison of the performance rates (TVR, TAR, TRR) within different study groups (by sample size, gender, age, heart rate) has been done with two-proportion Chi-squared test. A value of p≤0.05 is considered statistically significant.

#### 3.4.1. ECG lead configurations

The option to include any lead in the feature set (Eq 7) is used to train different LDA models for the following lead configurations, available in 12-lead ECG:

- Single leads: Li = [1, 2, …, or 12] for independent selection of leads (I, II, III, aVR, aVL, aVF, V1-V6);
- Limb leads: Li = [1:6] for joint selection of 6 limb leads (I, II, III, aVR, aVL, aVF);
- Chest leads: Li = [7:12] for joint selection of 6 chest leads (V1-V6);
- 12 ECG leads: Li = [1:12] for joint selection of all 12 leads (I, II, III, aVR, aVL, aVF, V1-V6).

#### 3.4.2. Test database size

Different subsets with all possible combinations of N = 10, 50, 100, 150, 200, 230 subjects within the total test database containing 230 subjects, are used to test the performance of the 12-lead LDA model. We note that the LDA model is taken exactly as trained on the independent training dataset with non-overlapping 230 subjects (valid also for the further tests 3–5).

#### 3.4.3. Gender

A number of 106 males (46%) and 124 females (54%) from the total population of 230 subjects in the test database are used to test the gender-specific performance of the LDA models for all single and multi-lead ECG configurations.

#### 3.4.4. Age

The test dataset with 230 subjects is divided into six age groups in respect of the subject’s age during session S1: <30 years (11 subjects), 30–39 years (16 subjects), 40–49 years (37 subjects), 50–59 years (86 subjects), 60–69 years (50 subjects), ≥70 years (30 subjects), used to test the age-related performance of the 12-lead LDA model.

#### 3.4.5. Heart rate (HR)

The test dataset with 230 subjects is divided into two kinds of groups to test the HR-related performance of the 12-lead LDA model:

- Groups based on the absolute HR value: The mean HR over 10s ECG in session S1 is used as a reference for defining five HR ranges: <60 bpm (26 subjects), 60–69 bpm (78 subjects), 70–79 bpm (76 subjects), 80–89 bpm (38 subjects), ≥90 bpm (12 subjects);
- Groups based on the absolute HR change: The absolute difference of the mean HR over 10s ECG in sessions S1 vs. S2 is used as a reference for defining three HR ranges: <10 bpm (143 subjects), 10–19 bpm (68 subjects), ≥20 bpm (19 subjects).

## 4. Results

### 4.1. Statistical analysis of the feature set

The first part of results is focused on statistical evaluation of the introduced QRS pattern matching features, trying to answer the question: “*Is there a statistical merit to use any of 12 ECG leads as a biometric modality*, *regarding high inter-subject differences (distinguishability) and low intra-subject differences (stability)*?”. In Table 1, the two groups of equal and different ID pairs are compared for all 12 leads, clearly indicating statistically different distributions (p<0.001):

- tEQU: the median value for the time equivalence between two QRS patterns is as high as 75–99% for equal IDs and as low as 53–74% for different IDs, with absolute difference in the range 18–31% points, considering all 12 leads.
- aDIF: the median value for the area difference between the two QRS patterns is as low as 0.2–10.8% for equal IDs and as high as 8–30.6% for different IDs, with absolute difference in the range 7.7–20.3% points, considering all 12 leads.

**Table 1:** Median value (quartile range) of tEQU and aDIF features for 12 ECG leads (S1 File). Statistically different distributions of 460 equal (IDS1 = IDS2) vs. 211140 different (IDS1≠IDS2) identity pairs are found in all leads (p<0.001).

	tEQU		aDIF	
Lead	ID_S1_ = ID_S2_	ID_S1_≠ID_S2_	ID_S1_ = ID_S2_	ID_S1_≠ID_S2_
**I**	93 (86–100)	65 (55–75)	1.8 (0–4.2)	14.2 (9.3–19.4)
**II**	96 (88–100)	65 (55–76)	0.9 (0–3.7)	12.9 (8.3–17.9)
**III**	75 (63–88)	48 (39–57)	10.6 (3.9–17.4)	30.6 (22.7–38.9)
**aVR**	99 (93–100)	74 (62–85)	0.2 (0–1.5)	8.0 (4.1–13.2)
**aVL**	76 (64–88)	50 (40–59)	9.0 (3.8–16.6)	29.3 (21.7–37.9)
**aVF**	85 (73–97)	56 (46–66)	4.5 (0.7–11)	20.5 (14.7–26.4)
**V1**	85 (72–98)	58 (48–68)	4.1 (0.4–9.1)	18.0 (12.3–23.8)
**V2**	75 (65–88)	53 (43–62)	9.4 (3.5–15.5)	24.2 (17.6–31.0)
**V3**	75 (63–88)	54 (44–63)	10.8 (3.8–18.9)	25.1 (18.3–32.1)
**V4**	82 (72–93)	60 (50–70)	6.5 (1.9–11.4)	18.3 (12.7–24.2)
**V5**	88 (77–98)	67 (56–77)	3.3 (0.5–8)	13.3 (8.5–18.5)
**V6**	88 (78–98)	70 (60–80)	3.5 (0.4–7.6)	11.2 (6.6–16.0)

### 4.2. Verification models in single and multi-lead configurations

This section presents a comparative study of the training and test performance of LDA verification models for different lead configurations, trying to answer the question: “*What is the optimal lead set for human biometrics*?”.

**Fig 4:**
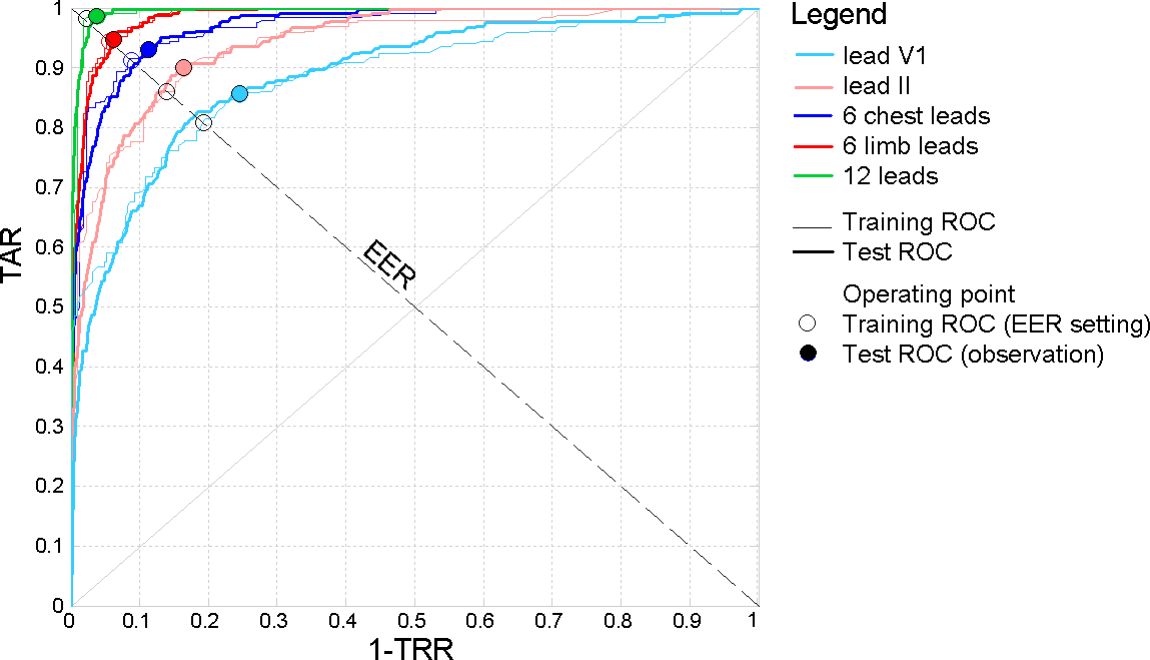
Training and test ROC curves of single and multi-lead ECG sets. The line EER (TAR = TRR) illustrates the choice of the operating point on the training ROC.

Table 2 shows the performance of lead-specific LDA verification models in terms of training and test AUC. The test AUC is found to be maximal for the single leads: II (0.941) among limb leads, V1 (0.885) among chest leads. The multi-lead sets are ranked in ascending order: 6 chest leads (0.97), 6 limb leads (0.986) and 12 leads (0.995). The respective ROC curves are illustrated in Fig 4. For each lead set, the observed good coincidence between training and test ROC curves (Fig 4) and the comparable training and test AUC values (Table 2) are a sign for confident training of the LDA model, which is able to adequately evaluate independent test data without a bias.

**Table 2:** Human verification performance of single and multi-lead ECG sets: AUC of the training and test ROC. The bolded values highlight the maximal AUC of the test-ROC for single limb leads, single chest leads, and the multi-lead sets.

	Limb leads						Chest leads						Multi-lead sets		
	I	II	III	aVR	aVL	aVF	V1	V2	V3	V4	V5	V6	Limb	Chest	12-leads
Train-AUC	.943	.937	.914	.909	.882	.887	.883	.877	.827	.829	.835	.796	.984	.968	.993
Test-AUC	.931	.**941**	.917	.924	.902	.927	.**885**	.869	.845	.875	.862	.799	.986	.970	**.995**

The settings of the optimal LDA model are defined for the training ROC operating point, which corresponds to balanced acceptance and rejection rates (TAR = TRR), commonly referred in the literature as the operating point at EER–see the ‘o’ mark in Fig 4. For the selected operating threshold LDA function, the observed performance on the independent test ROC could be considered as unbiased assessment of the human verification model–see the filled ‘o’ mark in Fig 4. The optimal LDA performance for both, training and test ROC operating points is reported in Table 3 for all types of lead sets. The training operating point behaves at EER (TAR = TRR), while the test operating point has a slight misbalance with TAR>TRR (difference of about 0.6% to 10% points), that is a natural consequence from the imbalanced test set with imbalance ratio (917:1) of different-to-equal ID pairs. The highlighted leads with maximal test set accuracy (Table 3) closely correspond to those with maximal test ROC AUC (Table 2).

**Table 3:** Human verification performance of single and multi-lead ECG sets for the EER operating point on the training ROC (Train-TAR = Train-TRR = Train-TVR). The observed performance on the independent test set has a slight bias Test-TAR>Test-TRR. The bolded values highlight the maximal TVR on the test set for single limb leads, single chest leads, and the multi-lead sets.

	Limb leads						Chest leads						Multi-lead sets		
	I	II	III	aVR	aVL	aVF	V1	V2	V3	V4	V5	V6	Limb	Chest	12-leads
Train-TVR (%)	87.4	86.1	84.4	83.7	81.1	80.4	80.9	80.0	74.4	76.1	75.0	73.9	94.4	91.3	98.0
Test-TAR (%)	86.5	90.0	84.4	85.7	83.9	87.4	85.7	81.3	80.0	84.4	81.7	69.6	94.8	93.0	98.7
Test-TRR (%)	84.1	83.6	81.3	85.1	80.8	80.4	75.5	78.9	72.5	73.7	74.1	74.1	93.8	88.8	96.3
Test-TVR (%)	85.3	**86.8**	82.8	85.4	82.3	83.9	**80.6**	80.1	76.2	79.0	77.9	71.8	94.3	90.9	**97.5**

**Fig 5:**
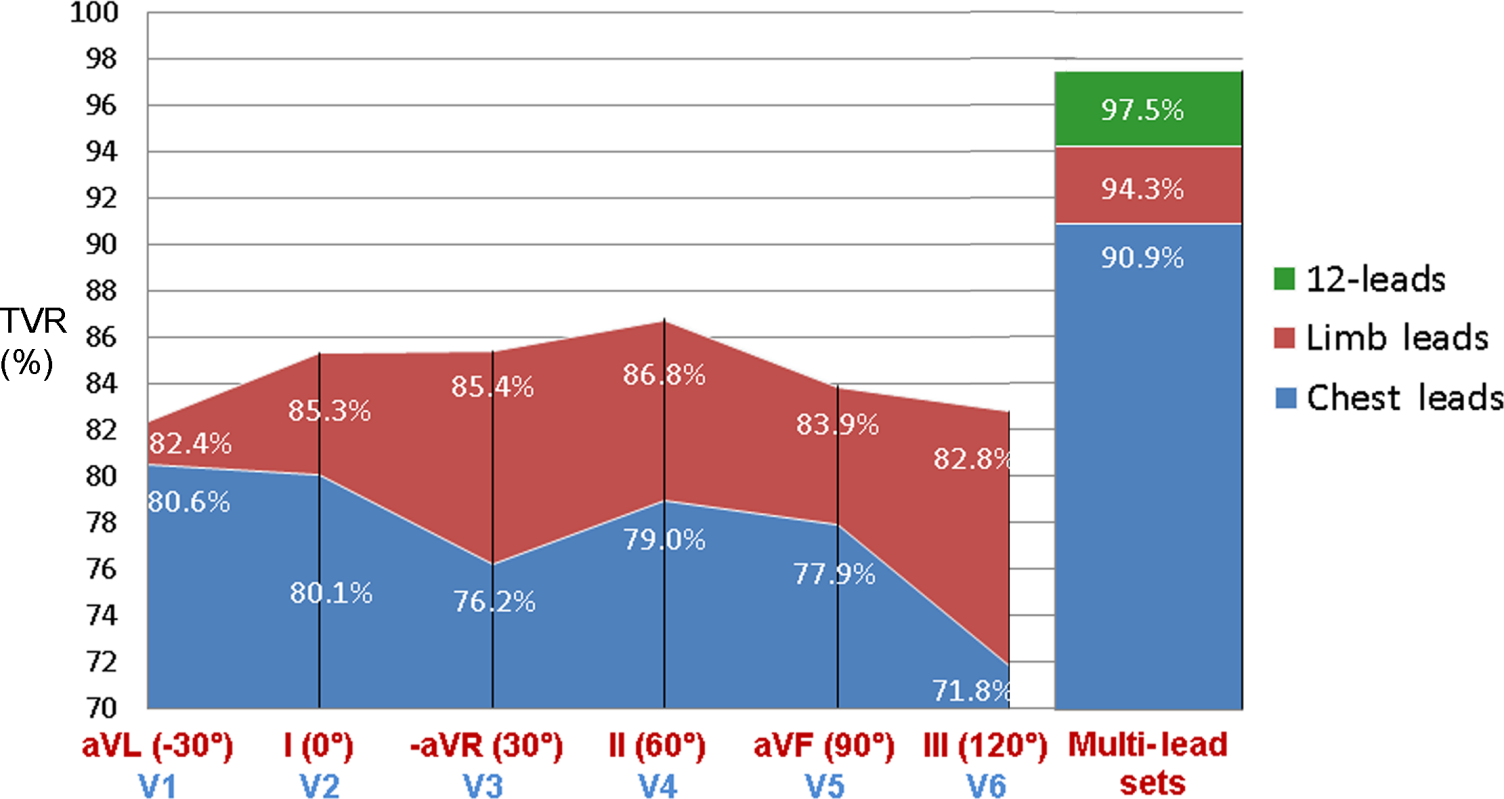
Test-TVR of single and multi-lead ECG sets. Single leads are ordered according to their spatial neighborhood, i.e. limb leads are presented in ascending order of their spatial angle in the frontal plane (given in brackets, from -30° to 120°); chest leads V1-V6 are presented according to their standard order in the horizontal plane.

Fig 5 provides graphical comparison of different lead sets in respect of their test-TVR. We observe maximal TVR profile of about (86.8–85.3%) for the limb leads (II, -aVR, I) within angles (60° to 0°) in the frontal plane. Other limb lead rotations (90°; 120°; -30°) decrease accuracy by (2.9%; 4%; 4.4%). The TVR profile of the chest leads is about 2% to 15% lower than limb leads, with decreasing trend from septal V1 (80.6%) to lateral V6 (71.8%). Here, we can rather distinguish anterior V3 (76.2%) with severe accuracy drop by 3.3% from the expected 79.5% as an approximation from its neighbors V2 (80.1%) and V4 (79%). The QRS pattern matching in multi-lead sets improves verification rate: chest (90.9%), limb (94.3%), 12-leads (97.5%).

### 4.3. Influence of the test database size and different physiological factors (gender, age, heart rate)

This section presents results in support of the stability of the LDA-based models’ performance, considering different factors that might influence the human verification process.

**Fig 6:**
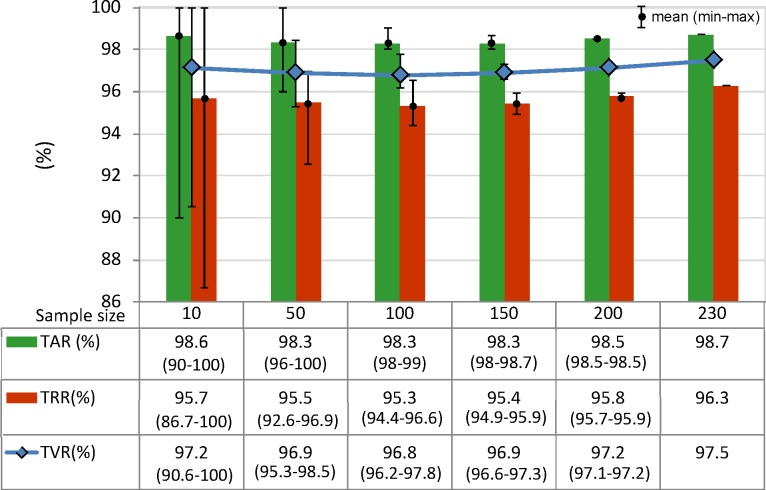
Performance of 12-lead LDA model in function of the number of subjects in the test database. TAR, TRR and TVR are reported as mean value (min-max range) after test of all possible combinations of 10, 50, 100, 150, 200, 230 subjects within the total test database with 230 subjects. The differences between groups are not statistically significant (p>0.05).

The influence of the test sample size is evaluated in Fig 6, regarding a broad range of subjects included in the test database (from 10 to 230 subjects). The 12-lead LDA model shows a stable performance with non-significant change of the mean value of all performance metrics (≤1%, p>0.67): TAR (mean value: 98.3–98.7%), TRR (95.3–96.3%), TVR (96.8–97.5%). We observe an inverse relationship between the sample size and the min-max margin of TAR, TRR, TVR values, i.e. the verification accuracy metrics might differ within a span up to 13.3%, 4.4%, 2.2%, 1%, <0.2%, depending on the selected combination of 10, 50, 100, 150, >200 subjects, respectively.

**Fig 7:**
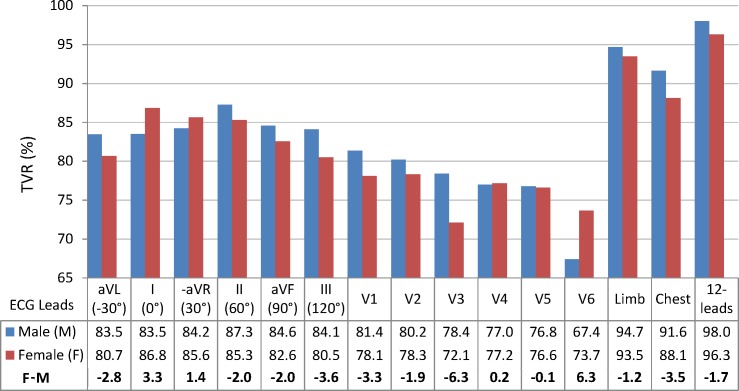
Gender-specific TVR performance of single and multi-lead LDA models, evaluated for 106 males and 124 females in the test database. For all leads, TVR (males vs. females) is not statistically significant (p>0.05).

The gender-specific performance of the LDA models for all single and multi-lead ECG configurations is evaluated in Fig 7. All TVR differences (males vs. females) are not significant (p>0.27). Better TVR for females are observed in the lateral leads V6 (by 6.3%), I (by 3.3%),–aVR (by 1.4%). Better TVR for males (by 1.9–3.6%) are observed in all other limb leads (aVL, II, aVF, III), chest leads V1, V2, emphasized in V3 (by 6.3%). The same TVR trend in favor of males is observed for the multi-lead ECG configurations, which is most prominent in the chest leads (by 3.5%) than in the limb leads (by 1.2%).

**Fig 8:**
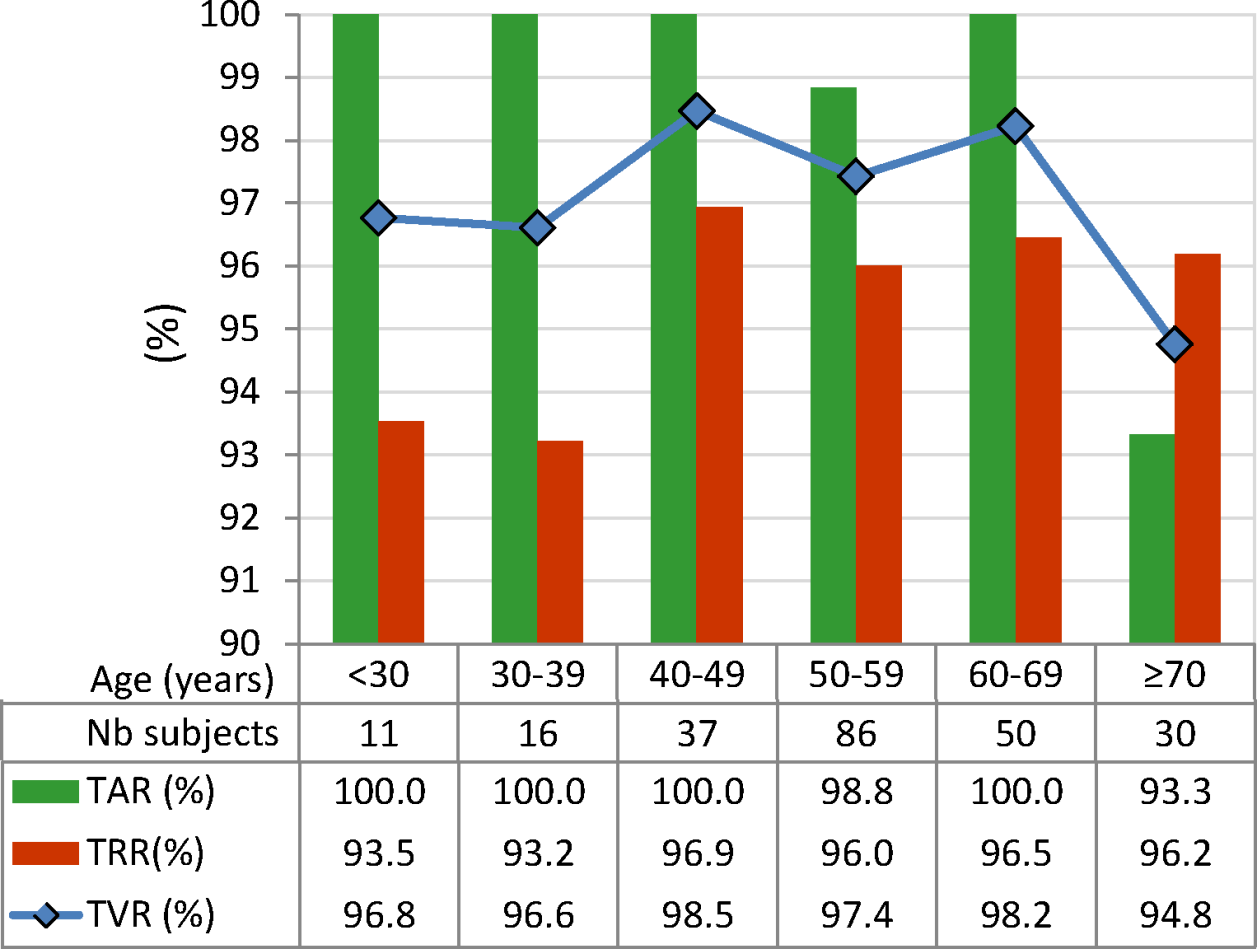
Age-specific performance of 12-lead LDA model, evaluated for 230 subjects in the test database, divided into six age groups. The differences between groups are not statistically significant (p>0.05).

The influence of the subject’s age is evaluated in Fig 8, regarding subjects covering a broad age ranges–from <30 years to ≥70 years old. The 12-lead LDA model shows a stable performance with non-significant change of all performance metrics (p>0.05). The most prominent drop in accuracy is observed for:

- The oldest group (≥70 years) vs. the younger group (60–69 years): TAR drops by about 6.7% (93.3% vs. 100%, p = 0.066). This results in TVR drop by about 3.7% (94.8% vs. 98.2%, p = 0.40).
- The youngest group (<40 years) vs. the older group (40–49 years): TRR drops by about 3.7% (93.2% vs. 96.9%, p = 0.54). This results in TVR drop by about 1.9% (96.6% vs. 98.5%, p = 0.66).

**Fig 9:**
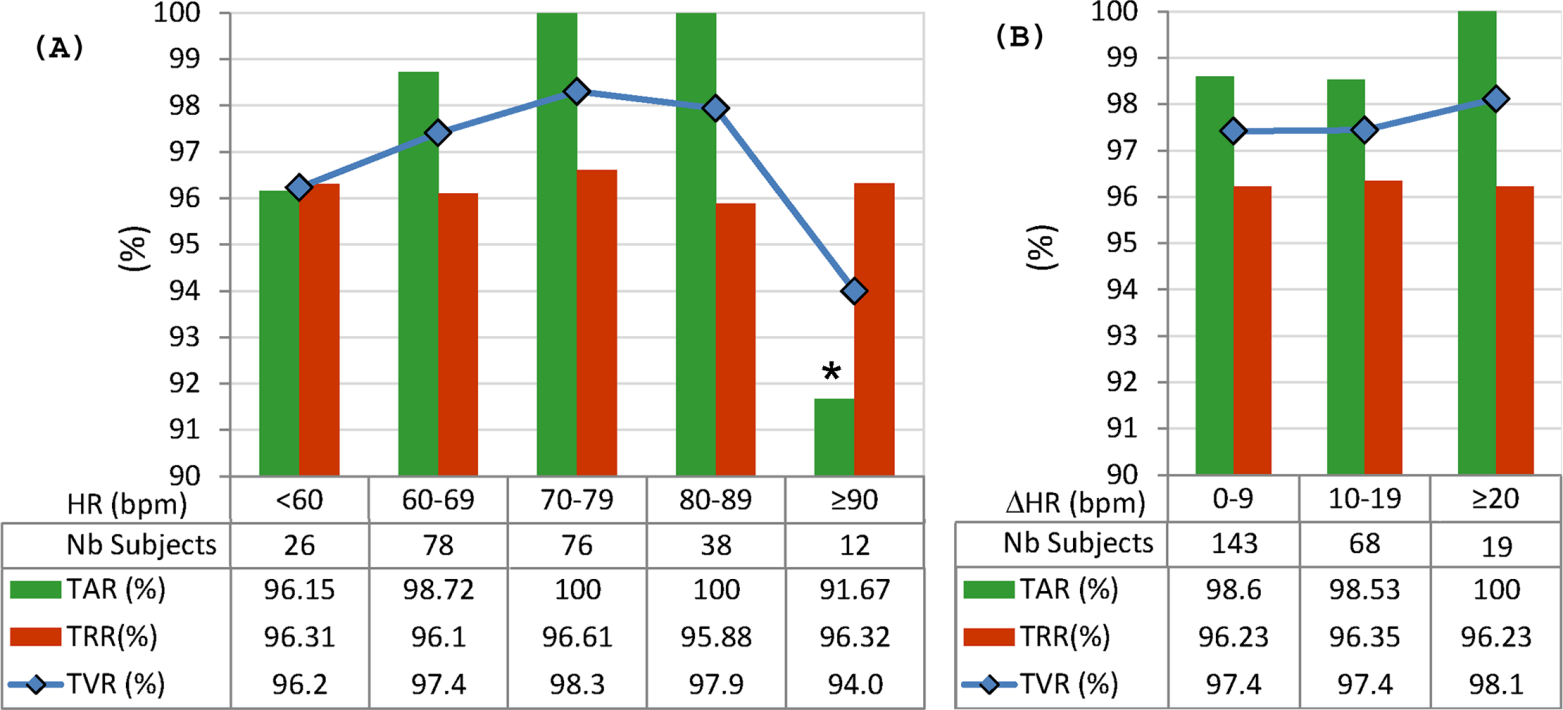
HR-specific performance of 12-lead LDA model, evaluated for 230 subjects in the test database, divided into: (A) 5 groups based on the absolute HR value in S1 session; (B) 3 groups based on the absolute HR change between S1 and S2 sessions (ΔHR). The differences between groups are not statistically significant (p>0.05), except TAR for ≥90 bpm (*p = 0.012).

The physiologically related HR differences between individuals (Fig 9A) and between different recording sessions of the same individual (Fig 9B) do not show to have great impact on the 12-lead LDA model performance. Both TRR (range 95.9–96.6%) and TVR (range 94–98.3%) keep stable (p>0.05) for the broad range of HR values (<60 bpm to ≥90 bpm), as well as for small (<10 bpm) and large (≥20 bpm) HR changes between the recording sessions. The same is valid for TAR (range 98.5–100% for HR = 60–89 bpm), with insignificant drop by 3.8% (96.15% vs. 100%, p = 0.087) for the slowest HR<60 bpm and significant drop by 8.3% (91.67% vs. 100%, p = 0.012) for the rapid HR≥90 bpm.

## 5. Discussion

This study reproduces a realistic scenario for the two-class person verification task, taking the decision to validate or to reject the subject identity based on binary QRS pattern matching between two 10s sessions with 12-lead ECG recordings. The presented cost-effective methodology uses a minimal feature set with only two straightforward QRS matching features per lead. Their statistical study on an uncommonly large population (460 subjects) proves a long-term stability within individuals (> 1 year basis) and distinguishability across individuals for any among 12 ECG leads (Table 1). We point out a confident LDA classification model with slight misbalance <3.5% between training and test accuracy reported on different datasets (Tables 2 and 3, Fig 4). The statistical analysis is presented for different perspectives of the human verification problem. First, we show the choice of the optimal ECG lead (Tables 2 and 3, Fig 5) for single (in the projection of lead II) and multi-lead scenario (limb leads and 12-leads); second, we show a stable performance without significant influence of the test database size (Fig 6) and different physiological factors–gender (Fig 7), age (Fig 8), heart rate (Fig 9). Finally in discussion, a comparison to other published results on human verification is presented, showing the competitive achievements in this study, especially in multi-lead ECG configurations (Table 4).

**Table 4:** Verification accuracy reported in published ECG biometric studies, which use at least two recording sessions per subject (distanced from days to years). Various accuracy metrics reported in other studies (EER, FAR, FRR, TAR, TRR) are transformed to the common metric TVR, using the direct conversions: TVR = 100-EER, TVR = (TAR+TRR)/2, TVR = 100-(FAR+FRR)/2.

Study	Database	Method	TVR
*Matos et al (2014)* [9]	10 subjects lead I (fingers)	STFT, symmetric relative entropy, log-likelihood ratio	86%
*Islam and Alajlan (2016)* [10]	112 subjects lead I (fingers)	PQRST template matching, Heart beat selection, Euclidean distance	87.2%
*da Silva et al (2013)* [8]	63 subjects lead I (fingers)	PQRST template matching, k-NN classifier, Euclidean, Cosine distance, SVM	85.6–90.9%
*Sriram et al (2009)* [13]	17 subjects, various activity conditions 1 limb lead	Autocorrelation, k-NN, Bayesian classifier, additional sensor (accelerometer)	84%
*Lourenço et al (2011)* [7]	16 subjects, (exercise) lead I (fingers)	Amplitudes of PQRST template, minimum Euclidean distance criterion	87%
*Agrafioti and Hatzinakos (2010)* [45]	52 subjects Lead I (wrist)	Autocorrelation, LDA	88%
*Odinaka et al (2010)* [46]	260 subjects, 1 lead (bilateral, lower rib cage)	STFT, Symmetric relative entropy, log-likelihood ratio	89% (one training session, 128 beats) 94% (two training sessions, 128 beats)
*Jekova and Bortolan (2015)* [25]	49 healthy subjects, 2 limb leads (I, II)	PQRST template matching, Cross-correlation	I: 80.3% II: 84.8% I+II: 87.2%
*Wübbeler et al (2007)* [43]	74 subjects, 3 limb leads	QRS template matching, 1^st^ and 2^nd^ temporal derivatives, minimum Euclidean distance	97.2%
*Tantawi et al (2013)* [37]	13 subjects (public PTB), 12-leads	Temporal and amplitude features, LDA, PCA, IGR, PASH	90.5–95.5%
*Jekova et al (2016)* [21]	574 healthy subjects, 12-leads	202 morphological features of PQRST, LDA	I: 64.4% 6 limb: 78% 6 chest: 80% 12-lead: 86%
*Krasteva et al (2017)* [22]	460 healthy subjects, 12-leads	PQRST template matching, Cross-correlation, LDA	II: 87.2% 6 limb: 92.7% 6 chest: 88.1% 12-lead: 94.9%
*Krasteva et al (2017)* [22]	460 healthy subjects, 12-leads	QRS template matching, Cross-correlation, LDA	II: 88.1% 6 limb: 91.6% 6 chest: 84.4% 12-lead: 93.8%
***This study***	460 healthy subjects, 12-leads	QRS pattern matching, LDA	II: **86.8%** 6 limb: **94.3**% 6 chest: **90.9**% 12-lead: **97.5**%

The milestones are further highlighted and discussed.

**Short-duration recording (10s)** is long enough to accumulate personalized average beat pattern with biometric significance, relying on the accurate beat extraction by a certified diagnostic ECG measurement and interpretation module (ETM, Schiller AG).

**Simple binary matching operations on 2D binary QRS matrices** are a cost-effective strategy for computation, using only AND and NAND operations applied to the small binary matrix *binQRS_Li_*(100*x*80), reserving a memory of about 1kB per lead. A minimal feature set with only two behavioral QRS pattern characteristics per lead is calculated, including:

- tEQU (calculated by binary AND operation) is a pattern similarity measure in the time scale (matching time)
- aDIF (calculated by binary NAND operation) is a pattern dissimilarity measure in the amplitude scale (mismatch area).

The use of normalized values for both metrics [0–100%] gives a subject invariant scale for pattern matching in large biometric databases. A simple visual biometric scheme is shown in Fig 3, where maximization of matching time and minimization of mismatch area in confident leads is a simple indicator for verification of patterns from the same subject (Fig 3, left panel), while the opposite distribution with short matching time and large mismatch area is a sign for dissimilar subjects (Fig 3, right panel). Such techniques for 2D binary computation, normalization and visualization are a cost-effective strategy for a biometric tool in smart portable devices that could optimally work with the minimal lead set, providing non-redundant and most reliable information.

**Long-term stability of the personalized QRS pattern in the presented time and amplitude matching scale** is statistically validated over a long period (> 1 year) across an uncommonly large population (460 subjects). We adopted two strategies against the measurement bias: (i) synchronous QRS pattern extraction in all 12-leads, using time-alignment to a single-lead reference pattern by maximal cross-correlation (Fig 2); (ii) time-amplitude approximation to mitigate the effect of intra-subject variations of the recording conditions across different sessions (Fig 3A, left panel), introducing an approximation tolerance of ±0.5% in the normalized amplitude scale and ±1ms in the time scale, as defined in Eq (2). Table 1 is a basis for tracking the long-term stability of the personalized QRS pattern in all 12-leads, showing large matching time tEQU = 75–99% median value (64–93% lower quartile) and low mismatch area aDIF = 0.9–10.8% median value (1.5–18.9% upper quartile) for 460 cases with ID_S1_ = ID_S2_. The statistical evaluation (median values tEQU/aDIF, %) highlights the leads with the most stable QRS patterns, ranked in the order: aVR (99/0.2), II (96/0.9), I (93/1.8) and those with the largest intra-subject instability: V3 (75/10.8), III (75/10.6), V2 (75/9.4), aVL (76/9.0), V4 (82/6.5), aVF (85/4.5), V1 (85/4.1), V6 (88/3.5), V5 (88/3.3). We speculate about technical and biological sources for the observed long-term QRS instability, i.e. changes of the recording conditions across different sessions and physiologically related intra-individual ECG variability. The relatively frequent human uncertainty about the proper landmarks of precordial leads (V1-V6) and the proximity to the signal source makes their QRS pattern sensitive to electrode misplacement errors [47–49]. Considering that limb leads are almost invariant to the actual positioning of the electrodes [27], we suggest about functional and physiological sources [50] for the observed instability of the inferior leads III, aVF (+90° to +120°) and the high lateral lead aVL (-30°).

**Unique personalized QRS patterns with distinctive time and amplitude matching measures across individuals** are statistically validated in a large population (211140 inter-subject pairs). Table 1 gives an evidence about relatively low matching time tEQU = 48–74% median value (59–85% upper quartile) and high mismatch area aDIF = 8–30.6% median value (4.1–22.7% lower quartile) after statistics of 12-lead QRS patterns in 211140 inter-subject pairs with ID_S1_≠ID_S2_. Comparing the groups of different-to-equal ID pairs, all leads have significantly distinguishable QRS matching features (p<0.001). Detailed review highlights the leads with the most distinctive QRS patterns across individuals in the time scale (II, I, aVF, III, V1 with the largest inter-to-intra subject reduction of the matching time by 27–31%), and in the amplitude scale (aVL, III with the largest inter-to-intra subject increase of the mismatch area by about 20%).

**Straightforward feature selection and optimization of binary LDA classifier** is achieved by ROC AUC maximization on the training dataset, which comprises the first half of subjects in the database (230 subjects). Unbiased validation of the LDA model is reported on the test set from the remaining data, fully independent on the training. As shown in Fig 4, both the training and test ROC curves are closely coinciding for the same LDA model, which is a straightforward value for reproducible performance that could be expected on other clinical data. Referring to ROC AUC as a statistic index that characterizes the overall predictive power of a binary classifier, unaffected by fluctuations caused by an arbitrarily chosen operating point with a trade-off between TAR and TRR [51, 52], the reported AUC values (Table 2) could rate the LDA verification model as ‘good’ (AUC = 0.8–0.9) for single chest leads and ‘excellent’ (AUC = 0.9–0.995) for single limb leads and all multi-lead configurations. The choice of the optimal LDA setting according to the EER strategy during training is consistent with a numerous human verification studies, which report equally weighted both errors from false verification and false rejection [7, 9, 10, 43, 45, 46]. In addition, our study validates LDA on independent test set (Table 3). Therefore, a slight misbalance of Test-TAR>Test-TRR (0.6–10% points) is considered as a consequence from the imbalance ratio (917:1) of different-to-equal ID pairs (see the shift of the test ROC operating point from the line TAR = TRR in Fig 4). The maximal drop in performance between Test-TVR vs. Train-TVR of about <3.5% (single leads) and <0.5% (all multi-lead sets), points out a confident LDA model.

**Objective selection of the optimal electrode scenario for ECG biometrics** is presented by comparative study of single limb-leads, single chest-leads and multi-lead configurations, extracted from clinical standard 12-lead ECG recordings, thus emulating a realistic case. The single-lead vector with the best biometric view over the personalized QRS pattern should present a trade-off between highest long-term stability (leads aVR, II, I as highlighted above) and highest distinctive matching across individuals (leads II, I, aVF, III, V1, aVL as highlighted above), thus justified for the common intersection (leads II, I). This hypothesis is confirmed by the LDA model performance (Tables 2 and 3) with maximal indices (Test-AUC, Test-TVR) observed for lead II (0.941, 86.8%) and slight accuracy drop for leads I (-0.01, -1.5%) and aVR (-0.017, -1.4%). This has a straightforward geometrical justification (Fig 5), which indicates that the frontal plane sector (60°-0°) encompassed by neighboring leads (II, -aVR, I) could be recognized as the most powerful projection of the cardiac vector for the aims of single-lead ECG human identity applications. The placement of the ECG electrodes on the chest is not recommendable because a gradual TVR drop from septal V1 (-6.2%) to lateral V6 (-15%) is observed in comparison to the limb lead II (Fig 5). The proximity to the signal source is not confirmed as an advantage for giving a view to unique personalized QRS patterns (only V1 has been highlighted above, however less distinctive than the limb leads). We rather suggest the major V1-V6 problem from the long-term instability of the QRS patterns, which are highly sensitive to electrode misplacement errors across the recording sessions. This effect has not been observed by Zhang and Wei [23], who underline that V1-V2 outperforms I-II by 5.5–10% in a human identification study. An explanation concerns the use of single-session recordings, not influenced by the real multi-session recording conditions.

We show that multi-lead identity systems could explore more detailed view of the subject-specific QRS patterns. Table 2 and Fig 5 indicate multi-lead TVR improvement up to 10.7% vs. the top-ranked single-lead II (86.8%). The test-TVR is reaching 90.9% for 6 chest leads, 94.3% for 6 limb leads and 97.5% for 12-lead ECG.

**Stable verification performance in respect to the test sample size and physiological factors (age, gender, HR)** is statistically proved using the test dataset with 230 subjects.

- Sample size: Fig 6 shows that small and large subsets (including from 10 to 230 individuals) are insignificantly influencing the performance metrics (TAR, TRR, TVR) of 12-lead LDA model with a mean value span <1% (p>0.67); however, caution should be paid on small datasets up to 50 individuals due to the observed large min-max margin of performance variation (up to 13.3% for 10 individuals down to 4.4% for 50 individuals), depending on the selected subset.
- Gender: Fig 7 shows that gender is not a significant factor in human biometrics with insignificant TVR differences by maximum of 6% for males vs. females (p>0.27). The largest differences are observed in chest leads V3 (6.3% in favor of men) and V6 (6.3% in favor of women), which are due to the failure in recognition of similar identity subjects. We suggest the human error for misplacement of V3 in women and V6 in men as the most probable reason for these errors. The better TVR in males for most of the leads (by 1.2–3.6% for II, III, aVL, aVF, V1, V2, all multi-lead sets) is due to the better recognition of different identity subjects. This is a normal consequence from the reported larger range of variation of the QRS amplitudes and durations in men than in women [53–55].
- Age: Fig 7 shows that the age is not a significant factor in human biometrics based on 12-lead ECG analysis. Insignificant failure for verification of the same identity subjects (TAR drop by 6.7%, p = 0.066) is observed in the oldest group (≥70 years old), suggested from the reported prevalence of aging-associated cardiovascular changes [56]. Insignificant failure for rejection of different identity subjects (TRR drop by 3.7%, p = 0.54) is observed in the youngest groups (<40 years), which implies that ECG morphology is less distinctive between younger individuals.
- HR: Fig 9 demonstrates that the proposed 12-lead QRS template matching model for human verification is robust to HR variations between individuals (covering HR range <90 bpm, Fig 9A) and HR changes between the recording sessions (covering the larges HR changes ≥20 bpm, Fig 9B). The largest problem is observed for verification of the same identity subjects with insignificant TAR drop by 2.8% for slow HR<60bpm and significant TAR drop by 8.3% for rapid HR≥90 bpm. This is an outcome from the reported heart rate dependency of the QRS duration with noticeable non-linear increase of QRS duration variations for heart rates >90 bpm [57].

**Comparative literature research** reveals wide variations of the ECG authentication accuracy, suggesting dependencies on the database size, experimental conditions, type and number of ECG leads, health status, etc. A comparison to other biometric studies is presented in Table 4, limited only to those under conditions similar to this study, i.e. two-class person verification classification, use of multi-session recordings. Most of the studies use private databases without public access; therefore we further give a reference to the accuracy results as originally published. Due to practical ECG acquisition simplification, major part of the studies employ single-lead configuration from lead I between fingers [7–10] or wrists [13, 45]. Based on different feature extraction and classification techniques, all above ‘lead I’ studies report TVR in the range from 84% to 88%, with one superior value of 90.9% for an SVM classifier [8]. We are suspicious about overtraining because all ‘lead I’ studies use the entire population for training or even training and test from different windows of the same recording in less than 20 subjects with limited intra-subject variation [7, 9, 13]. We report comparable TVR range for lead I (train-TVR = 87.4%, test-TVR = 85.3%), pretending for ‘unbiased’ validation on up to 40 times larger test population, independent from the training. Our finding for the optimal lead selection suggests a room for improvement of ‘lead I’ studies if the left arm finger/wrist electrode is moved on the body to form lead II equivalent. In bilateral lower rib cage configuration, Odinaka et al [46] reported the highest single-lead accuracy–about 89% or 94% if 128 beats from one or two training sessions are used, respectively. The latter training mode benefits from studying the impact of the long-term variability between the two sessions. Comparing Odinaka et al [46] and Matos et al [9] who implement the same signal-processing method for single-lead ECG biometrics (TVR = 89% vs. 86%), we might speculate that the electrode configuration and the good sticking of the ECG electrodes on the body [46] improves the accuracy compared to finger-based biometrics [9], largely susceptible to noise. We found three published studies, which investigate the feasibility of combined limb leads for human verification with reported TVR in a large span of about 20% points, i.e. minimal value of 78% with morphological PQRST features [21], 87.2% with PQRST cross-correlation [25] and 97.2% with Euclidean distance from the first and second QRS signal derivatives [43]. Our study, based on analysis of the same short QRS template, obtains about 3% lower TVR than the latter superior result. We see that [43] has not been verified on independent dataset and potentially might be over-trained to the empirical distance threshold of the whole population. Multi-lead ECG sets for human verification in configuration of only chest leads and 12-lead ECG is almost a blank area of research. There is evidence that the binary QRS template matching in this study outperforms morphological PQRST features (worsen by 2–7% [37], 11–22% [21]), cross-correlation PQRST matching (worsen by 1.7–2.6% for all multi-lead sets [22]) and cross-correlation QRS matching (worsen by 2.7–6.5% for all multi-lead sets [22]). We note that the comparison to our recent studies [21, 22] is straightforward because they use the same large biometric databases for training and validation.

The limitation of the study concerns the reported verification accuracy only on healthy (non-cardiac) individuals during rest. We might expect slight TAR reduction (failure to verify the same identity subject) in case of cardiovascular disease developed over time between the reference and test sessions, due to potentially affected ECG morphology, as suggested in [12, 25, 33, 41]. In such cases, the ECG biometric reference database might be permanently calibrated over years.

## 6. Conclusions

This study gives straightforward evidence about the questions:

- “*Is binary template matching able to capture significant 12-lead QRS pattern differences across individuals, while keeping stable personalized measurements in a long-term basis?”*
- “*How reliable are these differences seen from different leads in single- and multi-lead verification scenarios?*”
- “*Could we guarantee a stable biometric performance under different conditions, independent from the number of verified subjects, gender, age and heart rate?”.*

The justification of these questions is given by statistical validation on independent subset from a clinically relevant database across a large population, representative for physiologically related long-term ECG changes and multi-session recording conditions. The practical benefit of our findings is the presented cost-effective strategy for 2D binary computation, normalization and visualization as a biometric tool in smart portable devices. They can rely on an effective lead-selection scheme based on ranking of 12 ECG leads by maximal TVR. Our recommendations about the optimal electrode setting concern peripheral lead II (87%) in a single-lead scenario. Including one additional electrode on the left arm would increase TVR by 7.5%. The fusion of information from 6 more chest leads, forming the standard 12-lead ECG would increase TVR by additional 3%, reaching 97.5%–a verification accuracy, which is likely to be tolerated in commercial ECG biometric technologies with potential application for patient validation support and error screening of digital hospital databases. The individual ECG might be also a useful candidate as an add-on to improve established biometrical systems.

## Supporting information

S1 File The Archive contains all data related to the measurements of the pattern matching features in 12-lead ECG database, including all pairwise combinations between S1 and S2 sessions of the whole population, with clusterization to the subject’s identity (equal/different), data subset (training/test), age, gender, HR. (ZIP)
